# Racial disparities in children tested for SARS-CoV-2 at pediatric emergency departments: A prospective cohort study

**DOI:** 10.1093/pch/pxaf058

**Published:** 2025-08-09

**Authors:** Gabrielle Freire, Vikram Sabhaney, April Kam, Brett Burstein, Deepti Reddy, Richard J Webster, Kathleen Winston, Jason Emsley, Jocelyn Gravel, Robert Porter, Roger Zemek, Ahmed Mater, Marina I Salvadori, Simon Berthelot,, Darcy Beer, Naveen Poonai, Anne Moffatt, Bruce Wright,, Lianne J McLean, Stephen B Freedman

**Affiliations:** Division of Emergency Medicine, Department of Pediatrics, Faculty of Medicine, University of Toronto, Ontario, Canada; Child Health Evaluative Sciences, Peter Gilgan Centre for Research and Learning, The Hospital for Sick Children, Toronto, Ontario, Canada; Department of Pediatrics, Faculty of Medicine, University of British Columbia, Vancouver, British Columbia, Canada; British Columbia Children’s Hospital Research Institute, University of British Columbia, Vancouver, British Columbia, Canada; Department of Pediatrics, Faculty of Medicine, McMaster University, Hamilton, Ontario, Canada; Montreal Children’s Hospital, Division of Pediatric Emergency Medicine, McGill University Health Centre, and the Department of Biostatistics, Epidemiology and Occupational Health, McGill University, Montreal, Quebec, Canada; Clinical Research Unit, Children’s Hospital of Eastern Ontario Research Institute, Ottawa, Ontario, Canada; Clinical Research Unit, Children’s Hospital of Eastern Ontario Research Institute, Ottawa, Ontario, Canada; Department of Pediatrics, Cumming School of Medicine, University of Calgary, Calgary, Alberta, Canada; Department of Emergency Medicine, IWK Children’s Health Centre and QEII Health Sciences Centre, Dalhousie University, Halifax, Nova Scotia, Canada; Department of Pediatric Emergency Medicine, CHU Sainte-Justine, Université de Montréal, Montreal, Quebec, Canada; Janeway Children’s Health and Rehabilitation Centre, NL Health Services, St Johns, Newfoundland, Canada; Departments of Pediatrics and Emergency Medicine, University of Ottawa, Children’s Hospital of Eastern Ontario, Ottawa, Ontario, Canada; Section of Pediatric Emergency, Department of Pediatrics, Jim Pattison Children’s Hospital, University of Saskatchewan, Saskatoon, Saskatchewan, Canada; Public Health Agency of Canada, Ottawa, Ontario, Canada; Department of Pediatrics, McGill University, Montreal, Quebec, Canada; Departments of Family Medicine and Emergency Medicine, Laval University, Quebec City, Quebec, Canada; Department of Pediatrics and Child Health, The Children’s Hospital of Winnipeg, Children’s Hospital Research Institute of Manitoba, University of Manitoba, Winnipeg, Canada; Departments of Paediatrics, Internal Medicine, Epidemiology & Biostatistics, Children’s Hospital London Health Sciences Centre, Schulich School of Medicine & Dentistry, London, Ontario, Canada; Department of Paediatrics, Kingston Health Sciences Centre, Queen’s University, Kingston, Ontario, Canada; Department of Pediatrics and Women and Children’s Health Research Instituted, University of Alberta, Edmonton, Alberta, Canada; Division of Emergency Medicine, Department of Pediatrics, Faculty of Medicine, University of Toronto, Ontario, Canada; Departments of Pediatrics and Emergency Medicine, Cumming School of Medicine, University of Calgary, Calgary, Alberta, Canada

**Keywords:** SARS-CoV-2, Post-covid conditions, Race, Equity, Emergency medicine

## Abstract

**Objectives:**

To evaluate the association between race and SARS-CoV-2 test positivity and outcomes in children.

**Study Design:**

Secondary analysis of a prospective cohort study recruiting children < 18 years, tested for SARS-CoV-2 between August 2020 and February 2022, in Canadian pediatric emergency departments. Race was self-reported by participants. The primary outcome was SARS-CoV-2 test positivity. Secondary outcomes were medical interventions and hospitalization within 14 days of index visit, and post-COVID condition (PCC) at 90-day follow-up. Associations were evaluated using multi-level logistic regression models.

**Results:**

Seven thousand and two-thirty three children underwent SARS-CoV-2 testing; median age was 2.0 years (IQR: 1.0–5.0), and 3366 (46.5%) were female. 1440 (19.9%) children tested positive for SARS-CoV-2, 776 (10.7%) were hospitalized, and 153 (13.2%) test-positive children experienced PCC. Compared to White children, most racial minority groups were more likely to test positive for SARS-CoV-2 (Middle Eastern aOR [95% CI] 2.62 [2.07, 3.32], Black aOR 2.36 [1.85, 3.03], Latin American aOR 2.23 [1.58, 3.15], South Asian aOR 2.17 [1.67, 2.82], Indigenous aOR 2.09 [1.29, 3.37], Southeast Asian aOR 1.82 [1.27, 2.62], Multiracial aOR 1.35 [1.07, 1.69], and had lower odds of medical interventions. Only Indigenous children were at higher odds of hospitalization than White children (aOR [95% CI]: 2.48 [1.03, 5.95]). Black children were less likely to report PCCs than White children (aOR 0.44 [0.22–0.86]).

**Conclusions:**

Racial disparities exist in SARS-CoV-2 test positivity and outcomes among Canadian children seeking emergency care. A better understanding of the factors contributing to these differences is needed to promote equitable health across the population.

## INTRODUCTION

During the COVID pandemic, racial minority children were less likely to be tested for SARS-CoV-2, but more likely to test positive, be hospitalized, be admitted to the intensive care unit (ICU), and have higher mortality rates from SARS-CoV-2 infection (1,2). Multiple confounding factors may contribute to these associations including disparities in access to testing and medical assessments (3,4), pre-existing health conditions (e.g., obesity, diabetes) (1,5), and socioeconomic factors (e.g., location of living, parental occupation) (6–8).

Canadian studies have shown higher COVID-19 prevalence and mortality rates in neighborhoods with higher ethno-cultural composition (9), a higher likelihood of dying from COVID-19 among Black and South Asian populations (10), and higher seroconversion rates for children whose parents identify as non-White (11). Most Canadian studies did not specifically study children (9,10), and aggregated multiple racial minority groups (9–11), not seeking to identify differences between these groups.

The extent of COVID-19-related disparities among racial minority groups of children remains unclear. This study sought to determine the association between race and test positivity in a national sample of children tested for SARS-CoV-2 infection in Canadian pediatric emergency departments (EDs). Secondary objectives were to determine the association between race and: (1) medical interventions, (2) hospitalization, and (3) development of the post-COVID condition (PCC).

## METHODS

### Study design

This is a secondary analysis of a prospective observational surveillance project enrolling children tested for SARS-CoV-2 infection in one of 14 participating Canadian pediatric EDs who are members of Pediatric Emergency Research Canada (PERC), between August 4, 2020, and February 22, 2022. These EDs include all Canadian tertiary pediatric institutions located in urban centres across eight Canadian provinces (12). Provincial single-payer insurance systems cover all medically necessary hospital and physician services (13,14).

Research ethics board approval was obtained at each institution, and informed consent and assent procedures followed institutional policies. Data reporting follows the Strengthening the Reporting of Observational Studies in Epidemiology guideline (15).

### Participant enrollment

Eligible children, < 18 years old, were included if they underwent nasopharyngeal, nares or throat swab collection for SARS-CoV-2 nucleic acid testing. Children were excluded if they or their caregiver declined to participate, or if race data was missing or unspecified. The study protocol did not specify SARS-CoV-2 testing criteria, which was determined by local policies. Initially, testing was near universal but became more selective with the emergence of the Omicron variant. Research team members received a daily list of eligible participants, and contacted them by telephone, starting with the first child tested each day, to minimize the potential for selection bias and standardize recruitment.

### Data collection

We collected demographic (e.g., age, sex, race), clinical (e.g., days of illness, symptoms, symptom grouping, pre-existing medical conditions, vital signs) and epidemiological risk factors (e.g., attendance at group gathering or school/daycare, mask wearing) data from participants and/or their caregivers via interviews and/or self-administered electronic survey. Symptoms were recorded as dichotomous variables, and age was categorized into 0-5, 6-11, and 12-18 years. Mask wearing was self-reported, as adherence to public health recommendations may vary between racial groups (16–19). SARS-CoV-2 vaccination status was collected, but many participants were ineligible for vaccination at the time of enrollment (N = 6266, 87.2%, Supplemental Table 2) as vaccine approval occurred late into recruitment (20). Variant of concern (VoC) periods were assigned based on index visit date, and date ranges for SARS-CoV-2 variants are described elsewhere (21). Medical records were reviewed to extract data from the index ED visit (e.g., SARS-CoV-2 test result, other testing, treatments and outcomes) and determine ED re-visit and hospitalization within 14 days. Follow-up at 90 days was attempted via telephone or email to assess PCC presence.

Study procedures were standardized using a manual of operations and data was stored in a REDCap database.

### Exposure definitions

Race is a dynamic social construct shaped by sociopolitical forces (22,23). In medical reporting, race is based on perceived physical differences, while ethnicity refers to cultural background (e.g., language, religion, etc.) (22–26). Interpersonal and structural racism create inequities in healthcare access, quality, experiences, and outcomes (27). Therefore, we focused our analysis on race to evaluate these inequities (23–27).

Participants self-reported race data using Statistics Canada census survey options (27), and could select multiple groups. An “Other” option allowed free-text entries which were reviewed and re-categorized by two authors (G.F., D.R.) with adjudication from a third (V.S.) in case of disagreement. Data was mapped to Canadian Institute for Health Information (CIHI) standards into the following mutually exclusive categories: Black, East Asian, Indigenous, Latin American, Middle Eastern, Multiracial, South Asian, Southeast Asian and White (23,25,26,28). Participants who self-reported race using religion (e.g., “Muslim”) or country/region with mixed backgrounds (e.g., “Fiji,” “West Indies,” etc.), were categorized as “Other races” (Supplemental Table 1). Any racial group with fewer than 30 participants was collapsed with the “Other Races” group, following the Central Limit Theorem (29).

### Outcomes

Our primary outcome was SARS-CoV-2 test positivity on specimens collected in the ED or within 14 days. Secondary outcomes included receipt of medical intervention within 14 days of index visit, and, among children who tested positive, hospitalization within 14 days of index ED visit, and development of PCC at 90 days after index ED visit.

Medical interventions were analyzed as a composite outcome including chest radiograph, bloodwork, intravenous fluids, corticosteroids, or antibiotics. We describe the presence/absence of pneumonia on chest radiograph based on radiologist report (i.e., “definite pneumonia” or “consolidation”) (30). We describe results bloodwork results for CRP, D-dimer, lymphocyte and platelet counts, all associated with severe outcomes in children with SARS-CoV-2 (31).

PCC was identified if participants indicated new, persistent, or returning symptoms or health problems at 90 days, via multiple-choice or free-text response (32–35). Two authors (G.F., S.F.) independently analyzed free-text responses, blinded to SARS-CoV-2 test results, performing narrative review and grouping. A third reviewer (V.S.) resolved any disagreements.

### Statistical analyses

A multivariable binomial logistic regression model assessed SARS-CoV-2 test positivity by racial groups, combining negative and indeterminate results into one group. Covariates included demographic factors (i.e., age, sex), pre-existing conditions, attendance at social gatherings and school/daycare, mask wearing, duration of symptoms prior to presentation, VoC period, and ED site. Prematurity status and patient age interaction terms were explored but not significant. We evaluated receipt of medical interventions using the same multivariable regression model. We evaluated hospitalization and PCC with multivariate regression models adjusting for age, sex, and VoC period (for hospitalization), and age (for PCC). Given that the timing of the study limited vaccine eligibility and availability for our participants (Supplemental Table 2), we did not adjust for vaccination status.

Children who belong to multiple racial backgrounds are part of a heterogenous group with diverse healthcare experiences. Children identifying as White and a minority group may be perceived differently by healthcare providers than Multiracial children who are not partly White. There is no clear guidance on categorizing these participants in research. We conducted a sensitivity analysis following the Ontario Standards for the Identification, and Reporting of Systemic Racism (36), re-categorizing participants with mixed racial backgrounds, including White, to their minority group. Individuals who reported ≥ 2 non-White racial backgrounds remained classified as Multiracial. (Supplemental Figure 1).

For all analyses, we reported missing values for key variables. Missing values for symptom counts were treated as absent. Missing values for dependent variables were not imputed due to the likely violation of the missing-at-random assumption. Analyses were performed with R, version 4.4.2 (37).

## RESULTS

**Figure 1.**
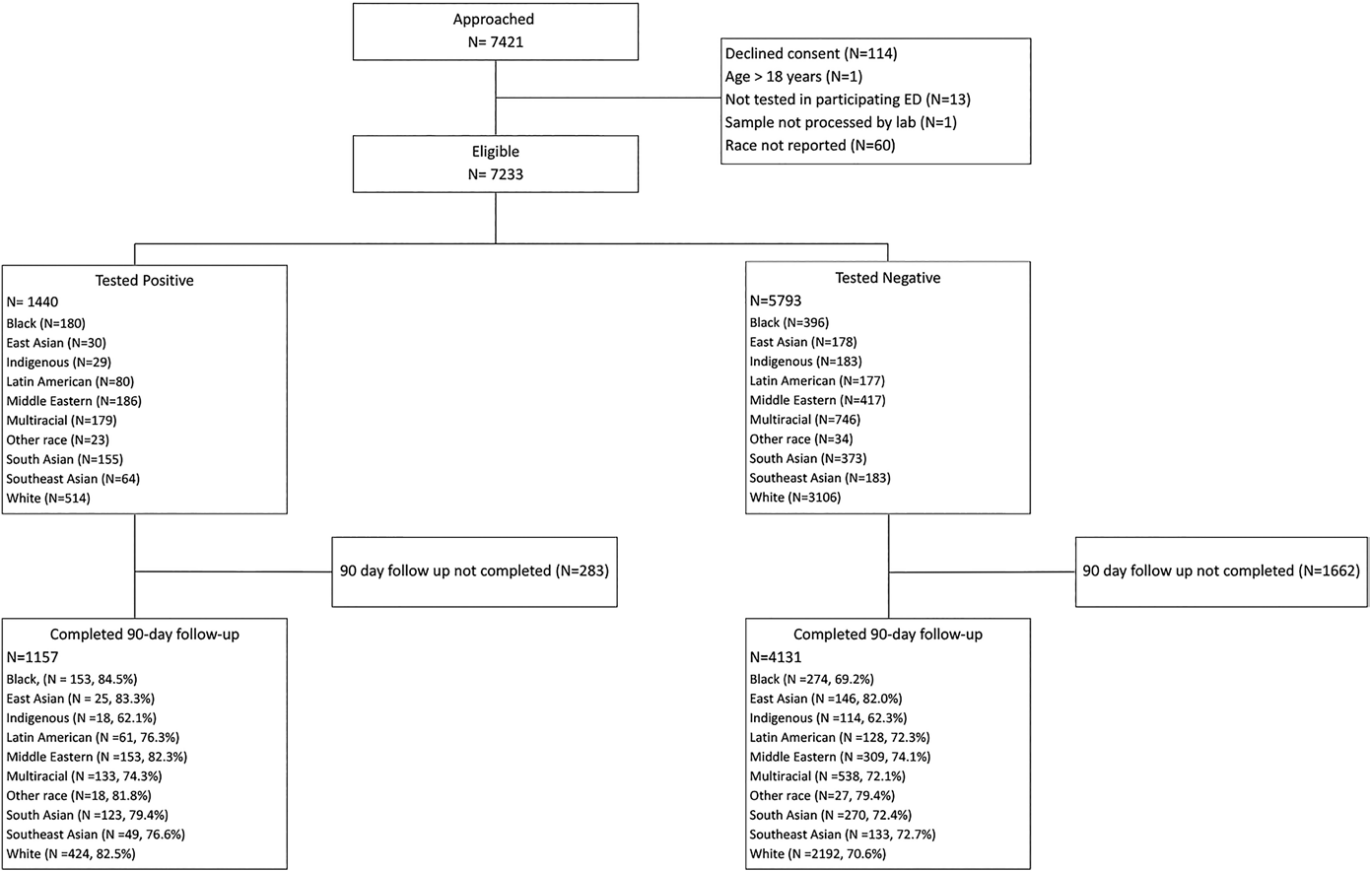
Flowchart of patients tested for SARS-CoV-2 at Canadian pediatric emergency departments between August 2020 and February 2022 (1,2). Participants who did not answer or stated that they preferred not to answer in their free-text entry were excluded from the cohort. Entries listed under “Other Race” included: Afghanistani, African, Asian, Canadian, Caribbean, Central Asian, Euro-Asian, Guyanese, Indo-Fijian, Israel, Jewish, Muslim, Pacific Islander, Tajikistani, Trinidad, Guyanese, Grenada, Turkish, Unknown Asian ancestry, Visible Minority, West Indian.

The study population included 7233 children < 18 years of age, tested for SARS-CoV-2 infection. Their median age was 2.0 years (IQR: 1.0–5.0, range: 0–17), 3366 (46.5%) were female, and 928 (12.8%) had a pre-existing medical condition. There were 3620 White (50.0%), 925 Multiracial (12.8%), 603 Middle Eastern (8.3%), 576 Black (8.0%), 528 South Asians (7.3%), 257 Latin American (3.6%), 247 Southeast Asian (3.4%), 212 Indigenous (2.9%), and 208 East Asian (2.9%) participants in our cohort. Forty-two (19.8%) Indigenous children had chronic medical conditions, 61 (28.8%) had a history of asthma or wheezing, and 36 (17.0%) had a prior pneumonia Figure 1, Table 1.

**Table 1.** Characteristics of children tested for SARS-CoV-2 in Canadian pediatric emergency departments between August 2020 and February 2022, by race

Variables	Black N = 576	East Asian N = 208	Indigenous N = 212	Latin American N = 257	Middle Eastern N = 603	Multiracial N = 925	Other race^*^ N = 57	South Asian N = 528	Southeast Asian N = 247	White N = 3620
Demographics										
Age (years): median (IQR) (min, max)	2.0 (1.0, 5.0) (0, 17)	2.0 (1.0, 5.0) (0.08, 16)	2.0 (0.9, 8.2) (0, 17)	2.0 (1.0, 6.0) (0, 17)	2.0 (1.0, 6.0) (0, 17)	2.0 (0.9, 5.0) (0, 17)	2.0 (0.9, 5.2) (0.8, 17)	2.0 (1.0, 4.2) (0, 17)	2.0 (1.0, 5.0) (0.08, 17)	2.0 (1.0, 5.0) (0, 17)
Sex: n (%)										
Male	311 (54.0%)	109 (54.2%)	107 (50.5%)	151 (58.8%)	314 (52.1%)	486 (52.5%)	29 (50.9%)	304 (57.6%)	146 (59.1%)	1910 (52.8%)
Female	265 (46.0%)	99 (47.6%)	105 (49.5%)	106 (41.2%)	289 (47.9%)	439 (47.5%)	28 (49.1%)	224 (42.4%)	101 (40.9%)	1710 (47.2%)
Health information; n (%)										
SARS-CoV-2 vaccination status at index visit										
Ineligible	4798 (83.2%)	182 (87.5%)	165 (77.8%)	215 (83.7%)	511 (84.7%)	814 (88.0%)	44 (77.2%)	467 (88.4%)	208 (84.2%)	3219 (88.9%)
Fully vaccinated	3 (0.5%)	1 (0.5%)	4 (1.9%)	7 (2.7%)	10 (1.7%)	9 (1.0%)	0 (0.0%)	4 (0.8%)	3 (1.2%)	52 (1.4%)
Partially vaccinated	5 (0.9%)	7 (3.4%)	3 (1.4%)	4 (1.6%)	3 (0.5%)	14 (1.5%)	1 (1.8%)	8 (1.5%)	3 (1.2%)	48 (1.3%)
Unvaccinated	75 (13.0%)	17 (8.2%)	31 (14.6%)	28 (10.9%)	71 (11.8%)	7 (8.0%)	12 (21.1%)	41 (7.8%)	31 (12.7%)	244 (6.7%)
Unknown	14 (2.4%)	1 (0.5%)	9 (4.2%)	3 (1.2%)	8 (1.3%)	14 (1.5%)	0 (0.0%)	8 (1.5%)	2 (0.8%)	57 (1.6%)
Past medical history										
Pneumonia	44 (7.6%)	14 (6.7%)	36 (17.0%)	26 (10.1%)	45 (7.5%)	92 (9.9%)	3 (5.3%)	36 (6.8%)	12 (4.9%)	422 (11.7%)
Asthma/wheezing	76 (13.2%)	31 (14.9%)	61 (28.8%)	56 (21.8%)	94 (15.6%)	191 (20.6%)	6 (10.5%)	88 (16.7%)	36 (14.6%)	814 (22.5%)
Asthma	41 (7.1%)	21 (10.0%)	43 (20.3%)	29 (11.3%)	55 (9.1%)	76 (8.2%)	3 (5.3%)	44 (8.3%)	24 (9.7%)	371 (10.2%)
Wheezing	35 (6.1%)	10 (4.8%)	18 (8.5%)	27 (10.5%)	39 (6.5%)	115 (12.4%)	3 (5.3%)	44 (8.3%)	12 (4.9%)	443 (12.2%)
Chronic conditions	69 (12.0%)	20 (9.6%)	42 (19.8%)	25 (9.7%)	58 (9.6%)	142 (15.4%)	10 (17.5%)	73 (13.8%)	27 (10.9%)	462 (12.8%)
Cardiovascular disease	8 (1.4%)	1 (0.5%)	9 (4.2%)	1 (0.4%)	13 (2.2%)	28 (3.0%)	1 (1.8%)	8 (1.5%)	5 (2.0%)	75 (2.1%)
Diabetes	1 (0.2%)	0 (0.0%)	1 (0.5%)	0 (0.0%)	0 (0.0%)	7 (0.8%)	1 (1.8%)	2 (0.4%)	0 (0.0%)	11 (0.3%)
Hematologic disease	29 (5.0%)	0 (0.0%)	1 (0.5%)	1 (0.4%)	6 (1.0%)	11 (1.2%)	4 (7.0%)	7 (1.3%)	2 (0.8%)	25 (0.7%)
Liver disease	1 (0.2%)	0 (0.0%)	0 (0.0%)	0 (0.0%)	2 (0.3%)	2 (0.2%)	0 (0.0%)	0 (0.0%)	0 (0.0%)	7 (0.2%)
Malignant neoplasm	0 (0.0%)	0 (0.0%)	2 (0.9%)	1 (0.4%)	2 (0.3%)	0 (0.0%)	0 (0.0%)	1 (0.2%)	2 (0.8%)	14 (0.4%)
Neurologic disease	10 (1.7%)	2 (1.0%)	14 (6.6%)	7 (2.7%)	12 (2.0%)	31 (3.4%)	2 (3.5%)	15 (2.8%)	5 (2.0%)	92 (2.5%)
Pulmonary disease	3 (0.5%)	1 (0.5%)	5 (2.4%)	2 (0.8%)	6 (1.0%)	15 (1.6%)	1 (1.8%)	5 (0.9%)	1 (0.4%)	45 (1.2%)
Renal disease	3 (0.5%)	4 (1.9%)	2 (0.9%)	2 (0.8%)	5 (0.8%)	8 (0.9%)	0 (0.0%)	12 (2.3%)	2 (0.8%)	45 (1.2%)
Rheumatologic disease	3 (0.5%)	1 (0.5%)	4 (1.9%)	0 (0.0%)	4 (0.7%)	3 (0.3%)	0 (0.0%)	2 (0.4%)	0 (0.0%)	32 (0.9%)
Other chronic disease	0 (0.0%)	0 (0.0%)	0 (0.0%)	0 (0.0%)	1 (0.2%)	2 (0.2%)	0 (0.0%)	0 (0.0%)	0 (0.0%)	12 (0.3%)
Behavioral or developmental disorders	2 (0.3%)	0 (0.0%)	1 (0.5%)	2 (0.8%)	2 (0.3%)	8 (0.9%)	0 (0.0%)	2 (0.4%)	0 (0.0%)	22 (0.6%)
Dermatologic disease	2 (0.3%)	7 (3.4%)	4 (1.9%)	3 (1.2%)	3 (0.5%)	17 (1.8%)	2 (3.5%)	11 (2.1%)	5 (2.0%)	25 (0.7%)
Endocrine disease	0 (0.0%)	0 (0.0%)	1 (0.5%)	1 (0.4%)	0 (0.0%	1 (0.1%)	0 (0.0%)	1 (0.2%)	2 (0.8%)	10 (0.3%)
Gastrointestinal disease	1 (0.2%)	0 (0.0%)	3 (1.4%)	2 (0.8%)	4 (0.7%)	13 (1.4%)	0 (0.0%)	9 (1.7%)	0 (0.0%)	54 (1.5%)
Genetic disease	0 (0.0%)	2 (1.0%)	1 (0.5%)	1 (0.4%)	1 (0.2%)	6 (0.6%)	1 (1.8%)	3 (0.6%)	1 (0.4%)	12 (0.3%)
HEENT disorder	1 (0.2%)	0 (0.0%)	1 (0.5%)	2 (0.8%)	1 (0.2%)	7 (0.8%)	0 (0.0%)	4 (0.8%)	2 (0.8%)	14 (0.4%)
Immunologic disease	1 (0.2%)	1 (0.5%)	2 (0.9%)	2 (0.8%)	3 (0.5%)	6 (0.6%)	0 (0.0%)	2 (0.4%)	0 (0.0%)	15 (0.4%)
Prematurity	67 (11.6%)	28 (13.5%)	35 (16.5%)	40 (15.6%)	69 (11.4%)	125 (13.%)	7 (12.3%)	78 (14.8%)	37 (15.0%)	465 (12.8%)
Epidemiological exposure risk factors^†^; n (%)										
Attendance at large social gatherings (N = 7218)	34 (5.9%) (n = 575)	15 (7.2%) (n = 207)	17 (8.1%) (n = 211)	27 (10.5%)	34 (5.7%) (n = 601)	91 (9.8%) (n = 924)	8 (14.0%)	27 (5.1%) (n = 525)	17 (6.9%)	329 (9.1%) (n = 3614)
School/daycare attendance (N = 7229)	333 (57.8%)	110 (53.1%) (n = 207)	78 (36.8%)	136 (52.9%)	302 (50.1%)	454 (49.1%)	28 (49.1%)	187 (35.5%) (n = 527)	88 (35.6%)	1985 (54.9%) (n = 3618)
Mask wearing (= 7231)										
Yes	170 (29.5%)	91 (44.0%) (n = 207)	95 (44.8%)	82 (31.9%)	193 (32.0%)	286 (30.9%)	25 (43.9%)	185 (35.1%) (n = 527)	97 (39.3%)	1250 (34.5%)
No	348 (60.4%)	85 (41.1%) (n = 207)	105 (49.5%)	148 (57.6%)	360 (59.7%)	541 (58.5%)	26 (45.6%)	289 (54.8%) (n = 527)	128 (581%)	2034 (56.2%)
Sometimes	58 (10.1%)	31 (15.0%) (n = 207)	12 (5.7%)	27 (10.5%)	50 (8.3%)	98 (10.6%)	6 (10.5%)	53 (10.1%) (n = 527)	22 (8.9%)	336 (9.3%)

^*^Entries listed under “Other Race” included: African, Asian, Canadian, Caribbean, Central Asian, Euro-Asian, Guyanese, Indo-Fijian, Israel, Jewish, Muslim, Pacific Islander, Tajikistani, Trinidad, Guyanese, Grenada, Turkish, Unknown Asian ancestry, Visible Minority, West Indian;

^†^Attendance at in-person school or daycare and attendance at large social gatherings were asked as yes/no questions. Mask use was asked as never/sometimes/always

### Outcomes

**Figure 2.**
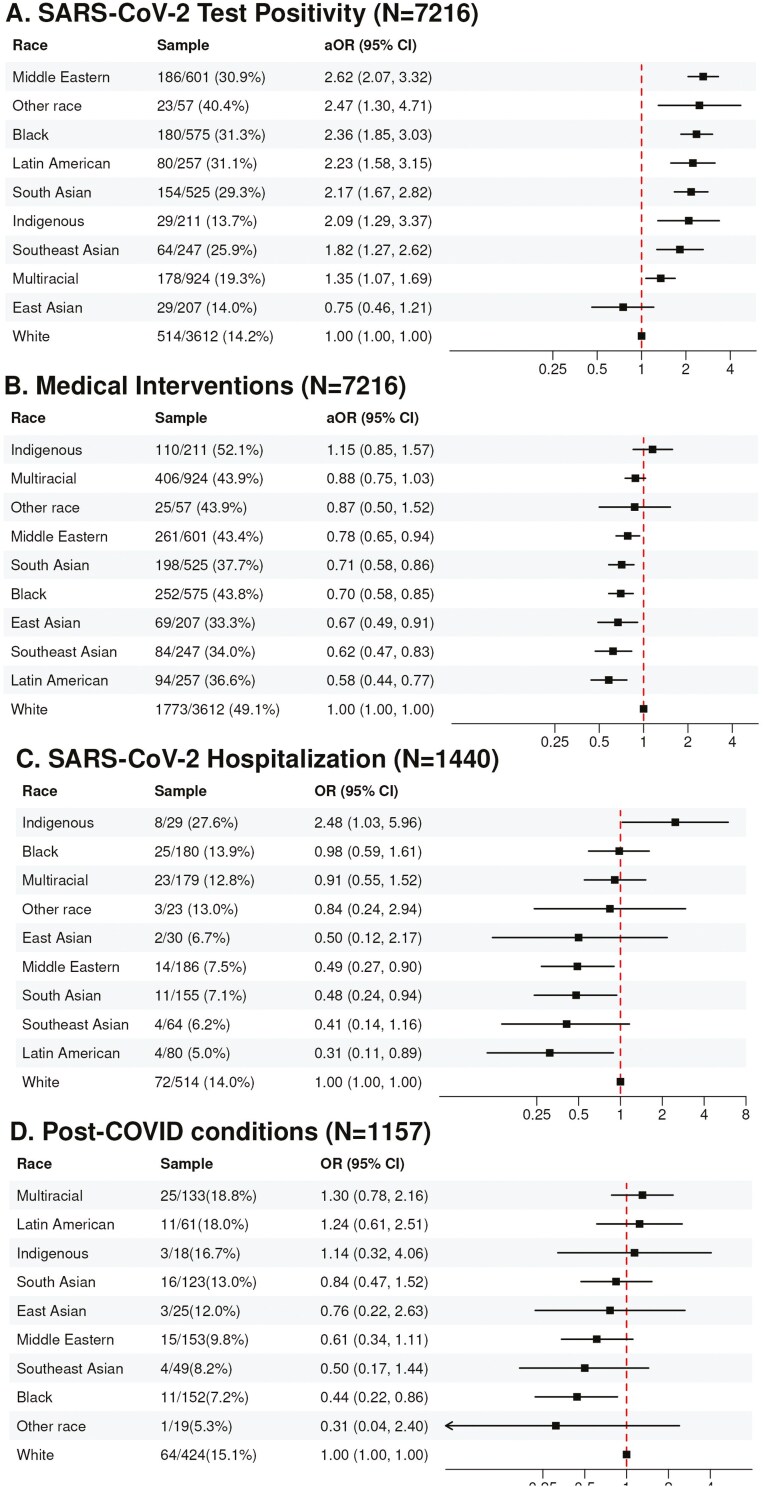
Forest Plot demonstrating the odds of SARS-CoV-2 clinical outcomes for children of various racial backgrounds tested for SARS-CoV-2 in Canadian pediatric emergency departments between August 2020 and February 2022 (1,2). Results for test positivity and medical interventions were obtained using multivariate regression model adjusting for age, sex, pre-existing conditions, attendance at social gatherings and school/daycare, mask wearing, duration of symptoms prior to presentation, VoC period, and ED site. Results for hospitalization were obtained using a multivariate regression model adjusting for age, sex, and ED site. Results for PCC were obtained using multivariate regression model adjusting for age. Participants were excluded from the test positivity and medical intervention models due to missing values for the following covariates: attendance at large social gatherings (n = 15), attendance at in-person school (n = 4), mask wearing (n = 2), symptom duration (n = 1).

Overall, 1440 (19.9%) children tested positive for SARS-CoV-2, 776 (10.7%) were hospitalized, and 41 (0.6%) were admitted to an ICU. Of children with SARS-CoV-2, 153 (13.2%) experienced PCC; Figure 1, Table 2. Eight out of nine racial minority groups were more likely to test positive compared to White children (aOR [95%CI] 1.35 [1.07-1.69] to 2.62 [2.07-3.32]); the only racial group to not differ was East Asian children. All racial minority children were less likely to receive medical interventions (aOR [95%CI] 0.58 [0.44–0.77] to 0.78 [0.65–0.94]), except for Indigenous (aOR 1.15 [0.85–1.57]), Multiracial (aOR 0.88 [0.75–1.03]), and other race (aOR 0.87 [0.50-1.52]) children. Only Indigenous children were more likely to be hospitalized than White children (aOR 2.48 [1.03-5.96]) in the primary analysis. Black children were less likely to experience PCCs than White children (aOR 0.44 [0.22–0.86]); Figure 2, Table 2. Other racial minority children were as likely to experience PCCs as White children; Figure 2, Table 2.

**Table 2.** Clinical presentation of children tested for SARS-CoV-2 in Canadian pediatric emergency departments between August 2020 and February 2022, by race

	Black N = 576	East Asian N = 208	Indigenous N = 212	Latin American N = 257	Middle Eastern N = 603	Multiracial N = 925	Other race N-57^*^	South Asian N = 528	Southeast Asian N = 247	White N = 3620
Symptom duration (days); median (IQR)	3.0 (1.0, 5.0)	2.0 (0.0, 4.0)	2.0 (1.0, 5.0)	2.0 (1.0, 4.0)	3.0 (2.0, 4.0)	3.0 (1.0, 5.0)	3.0 (1.0, 5.0)	2.0 (1.0, 4.0)	2.0 (1.0, 4.0)	3.0 (1.0, 5.0)
Number of symptoms; n (%)										
None	32 (5.%)	6 (2.9%)	7 (3.3%)	8 (3.1%)	30 (5.0%)	21 (2.3%)	4 (7.0%)	17 (3.2%)	8 (3.2%)	119 (3.3%)
<5	277 (48.1%)	114 (54.8%)	69 (32.5%)	103 (40.1%)	287 (47.6%)	319 (34.5%)	20 (35.1%)	244 (46.2%)	136 (55.1%)	1448 (40.0%)
≥5	267 (46.4%)	88 (42.3%)	136 (64.2%)	146 (56.8%)	286 (47.4%)	585 (63.2%)	33 (57.9%)	267 (50.6%)	103 (41.7%)	2053 (56.7%)
Triage vitals at index visit; mean (IQR)										
Temperature (N = 6831)	37.4 (36.9, 38.2) (N = 52)	37.4 (36.8, 38.1) (N = 19)	36.9 (36.6, 37.5) (N = 211)	37.2 (36.7, 38.0) (N = 245)	37.4 (36.8, 38.2) (N = 546)	37.2 (36.8, 38.0) (N = 871)	37.3 (36.7, 378.) (N = 56)	37.1 (36.7, 37.8) (N = 509)	37.3 (36.8, 38.1) (N = 23)	37.2 (36., 38.0) (N = 3434)
Pulse (N = 6857)	131 (114, 150) (N = 53)	133 (117, 152) (N = 199)	129 (111, 148) (N = 209)	132 (111, 151) (N = 243)	132 (111, 151) (N = 548)	132 (113, 153) (N = 873)	126 (107, 144) (N = 55)	136 (118, 155) (N = 507)	132 (116, 156) (N = 23)	132 (110, 153) (N = 3454)
Systolic blood pressure (N = 3503)	105 (97, 117) (N = 256)	104 (95, 116) (N = 102)	108 (98, 121) (N = 128)	103 (94, 115) (N = 140)	106 (98, 117) (N = 267)	106 (97, 115) (N = 460)	110 (95, 115) (N = 27)	105 (95, 114) (N = 273)	106 (98, 114) (N = 137)	107 (98, 117) (N = 1713)
Respiratory rate (N = 6554)	28 (22, 36) (N = 488)	30 (24, 36) (N = 195)	26 (22, 32) (N = 201)	28 (24, 36) (N = 233)	28 (22, 36) (N = 494)	28 (24, 36) (N = 850)	28 (24, 36) (N = 55)	28 (24, 36) (N = 497)	28 (22, 36) (N = 230)	28 (22, 36) (N = 3311)
Oxygen saturation (N = 6583)	99.0 (98.0, 100.0) (N = 520)	99.0 (97.0, 100.0) (N = 185)	98.0 (97.0, 99.0) (N = 210)	98.0 (98.0, 100.0) (N = 231)	98.0 (98.0, 100.0) (N = 522)	98.0 (97.0, 100.0) (N = 827)	98.0 (98.0, 100.0) (N = 51)	98.0 (97.0, 100.0) (N = 491)	99.0 (97.0, 100.0) (N = 233)	98.0 (97.0, 99.0) (N = 3313)
Type of symptoms; n (%)										
Any systemic symptom	403 (69.8%)	174 (82.9%)	176 (83.0%)	211 (82.1%)	451 (74.8%)	770 (83.2%)	41 (73.2%)	422 (79.9%)	190 (77.6%)	2871 (79.3%)
Any respiratory symptom	416 (72.2%)	120 (57.7%)	162 (76.4%)	180 (70.0%)	395 (65.5%)	682 (73.7%)	44 (77.2%)	355 (67.2%)	157 (63.6%)	2600 (71.8%)
Any gastrointestinal symptom	252 (43.8%)	95 (45.7%)	104 (49.1%)	135 (52.5%)	291 (48.3%)	455 (49.2%)	25 (43.9%)	253 (47.9%)	109 (44.1%)	1641 (45.3%)
Any neurologic symptom	120 (20.8%)	39 (18.3%)	77 (36.3%)	67 (26.1%)	143 (23.7%)	221 (23.9%)	11 (19.3%)	131 (24.8%)	55 (22.3%)	843 (23.3%)
Loss of smell (N = 5523)	9 (1.9%) (N = 482)	1 (0.6%) (N = 161)	5 (2.9%) (N = 172)	4 (1.9%) (N = 213)	14 (2.8%) (N = 492)	10 (1.4%) (N = 704)	1 (2.3%) (N = 43)	9 (2.1%) (N = 424)	4 (2.1%) (N = 193)	25 (0.9%) (N = 2639)
SARS-CoV-2 test result										
Positive	180 (31.2%)	30 (14.4%)	29 (13.7%)	80 (31.1%)	186 (30.8%)	179 (19.4%)	23 (40.4%)	155 (29.4%)	64 (25.9%)	514 (14.2%)
Negative	395 (68.6%)	178 (85.6%)	182 (85.8%)	175 (68.1%)	413 (68.5%)	736 (79.6%)	34 (59.6%)	369 (69.9%)	183 (74.1%)	3099 (85.6%)
Indeterminate	1 (0.2%)	0 (0.0%)	1 (0.5%)	2 (0.8%)	4 (0.7%)	10 (1.1%)	0 (0.0%)	4 (0.8%)	0 (0.0%)	7 (0.2%)
Variant of Concern; n (%)										
Wild type	21 (36.6)	93 (44.7.%)	97 (45.8%)	122 (47.5%)	255 (42.3%)	411 (44.4%)	25 (43.9%)	225 (42.6%)	112 (45.3%)	1853 (51.2%)
Alpha	97 (16.8%)	47 (22.%)	53 (25.0%)	38 (14.8%)	116 (19.2%)	188 (20.3%)	9 (15.8%)	112 (21.2%)	41 (16.6%)	548 (15.1%)
Delta	190 (33.0%)	49 (23.6%)	51 (24.1%)	70 (27.2%)	169 (28.0%)	229 (24.8%)	17 (29.8%)	148 (28.0%)	69 (27.9%)	828 (22.9%)
Omicron	79 (13.7%)	19 (9.1%)	11 (5.2%)	27 (10.5%)	63 (10.4%)	97 (10.5%)	6 (10.5%)	43 (8.1%)	25 (10.1%)	391 (10.8%)
Medical interventions; n (%)										
Chest radiograph	86 (14.95.1.5.1)	17 (8.2%)	29 (13.7%)	31 (12.1%)	81 (13.4%)	108 (11.7%)	8 (14.0%)	55 (10.4%)	23 (9.3%)	532 (14.7%)
Bloodwork	130 (22.6%)	32 (15.4%)	45 (21.2%)	46 (17.9%)	131 (21.7%)	169 (18.3%)	15 (26.3%)	102 (19.3%)	39 (15.8%)	759 (21.0%)
CRP (N = 983)	5.0 (1.2, 15.5) (N = 74)	13.6 (5.0, 39.0) (N = 21)	9.6 (4.0, 40.5) (N = 23)	11.1 (1.9, 32.4) (N = 28)	9.2 (2.7, 34.8) (N = 92)	6.0 (2.2, 28.1) (N = 115)	14.7 (0.6, 43.8) (N = 10)	5.5 (2.2, 29.6) (N = 77)	7.1 (1.8, 34.1) (N = 28)	5.4 (2.5, 25.5)(N = 515)
D-dimer (N = 65)	869.5 (593.5, 1351.0) (N = 4)	- (N = 0)	289.0 (289.0, 289.0) (N = 1)	775.0 (628.8, 1130) (N = 4)	1110.0 (760.0, 2200.0) (N = 3)	840.0 (475.0, 1730.0) (N = 9)	3230.0 (3230.0, 3230.0) (N = 1)	1226.5 (525.5, 1715.8) (N = 6)	85.8 (143.7 427.9) (N = 2)	570.0 (243.0, 1478.5) (N = 35)
Lymphocyte (N = 1341)	2900.0 (1775.0, 4210) (N = 120)	2300.0 (1335.0, 3860.0) (N = 31)	2680.0 (1600.0, 3630.0) (N = 45)	2000.0 (1320.0, 3490.0) (N = 45)	3100.0 (1550.0, 4880.0) (N = 123)	2900.0 (1500.0, 4260.0) (N = 161)	2650.0 (1365.0, 4177.5) (N = 12)	3025.0 (1547.5, 4975.0) (N = 98)	3200.0 (2820., 4790.0) (N = 3)	2588.5 (1472.5, 4400.0) (N = 670)
Platelet	288.0 (230.0, 386.0) (N = 129)	305.0 (235.0, 346.0) (N = 30)	359.0 (300.0, 458.0) (N = 45)	285.0 (214.2, 339.8) (N = 46)	297.5 (242.0, 357.8) (N = 130)	331.0 (246.0, 413.5) (N = 167)	290.5 (252.2, 335.0) (N = 14)	286.0 (229.5, 370.2) (N = 100)	33.0 (262.0, 405.5) (N = 3)	292.0 (230.2, 375.8) (N = 754)
Intravenous fluids	62 (10.%)	10 (4.8%)	22 (10.4%)	20 (7.8%)	42 (7.0%)	76 (8.2%)	4 (7.0%)	32 (6.1%)	15 (6.1%)	296 (8.2%)
Steroids administered	50 (8.7%)	20 (9.6%)	42 (19.8%)	25 (9.7%)	55 (9.1%)	122 (13.2%)	5 (8.8%)	50 (9.5%)	19 (7.7%)	572 (15.8%)
Antibiotics administered	104 (18.1%)	25 (12.0%)	40 (18.9%)	37 (14.4%)	94 (15.6%)	144 (15.6%)	10 (17.5%)	70 (13.3%)	33 (13.4%)	610 (16.9%)
Any intervention received	253 (43.9%)	70 (33.7%)	110 (51.9%)	94 (36.6%)	262 (43.4%)	407 (44.0%)	25 (43.9%)	200 (37.9%)	84 (3.9)	1775 (49.0%)
Level of care^†^; n (%)										
Hospitalized	68 (11.8%)	12 (5.8%)	32 (15.1%)	23 (8.9%)	54 (9.0%)	100 (10.8%)	8 (14.0%)	50 (9.5%)	20 (8.1%)	409 (11.3%)
Return ED visits within 14 days of index visit for test-positive participants										
At least one return visits	14 (9.0%)	3 (10.7%)	2 (9.5%)	7 (9.2%)	13 (7.6%)	20 (12.8%)	2 (10.5%)	11 (7.6%)	2 (3.3%)	60 (13.6%)
Post-COVID condition; n (%)										
PCC reported (N = 1157)	11 (7.2%) (N = 152)	3 (12.0%) (N = 25)	3 (16.7%) (N = 18)	11 (18.0%) (N = 61)	15 (9.8%) (N = 153)	25 (18.8%) (N = 133)	1 (5.3%) (N = 19)	16 (13.0%) (N = 123)	4 (8.2%) (N = 49)	64 (15.1%) (N = 424)

^*^Entries listed under “Other Race” included: African, Asian, Canadian, Caribbean, Central Asian, Euro-Asian, Guyanese, Indo-Fijian, Israel, Jewish, Muslim, Pacific Islander, Tajikistani, Trinidad, Guyanese, Grenada, Turkish, Unknown Asian ancestry, Visible Minority, West Indian;

^†^Admission to hospital includes admission to an observational unit

### Sensitivity analysis

Overall, 708 children (9.8%) were re-assigned into their respective racial minority groups. Results of the sensitivity analysis were consistent with the primary analysis for medical interventions and PCCs, while they differed for test positivity, and hospitalization. In the sensitivity analysis, six racial minority groups were more likely to test positive, compared to White children. Differences were now tempered (from increased odds to no difference) for Indigenous and Other race children. For hospitalizations, no racial minority groups were more likely to be hospitalized; results were now tempered from increased odds to no difference for Indigenous children; Supplemental Figure 2.

## DISCUSSION

This is the first study to report racial disparities in care and outcomes at Canadian pediatric EDs. It is also the first prospective cohort of children tested for SARS-CoV-2, using self-reported race data. Compared to White children, most racial minority children were more likely to test positive for SARS-CoV-2, but less likely to receive medical interventions. Among children who tested positive for SARS-CoV-2, only Indigenous children had higher hospitalization rates than White children, and only Black children had lower odds of PCC compared to White children.

While previous studies have reported that outcomes vary between racial groups (1,2), most used EMR-recorded race data, which is less accurate than our self-reported data (24,25,28). Our findings also differ slightly: 1) we were able to identify specific racial minority groups at higher odds of testing positive for SARS-CoV-2, 2) we found that most racial minority groups were not at increased risk of hospitalization, and 3) no racial minority groups were at increased risk of PCCs.

Our finding that children of most racial minority groups were more likely to test positive is consistent with literature from the United States (2,38), United Kingdom (1,39), and Europe (40). This may be due to lower testing rates in racial minority children (1,3); whereby those tested may have been more symptomatic, driving higher test positivity rates. Children of racial minority groups may also be more likely to test positive for SARS-CoV-2 by living with adults who are employed in jobs with high potential for SARS-CoV-2 exposure (41,42). Cultural elements (e.g., multi-generational households) and lower socioeconomic status in some racial minority groups (43), can lead to crowded housing which facilitates virus transmission. Language barriers and a lack of scientific trust among racial minority families may also have limited their ability to understand, or comply with, public health infection prevention measures, contributing to higher infection rates (16–19,44,45).

Previous studies have described racial disparities in diagnostic testing and treatment for pediatric conditions like bronchiolitis (46), gastroenteritis (47), and respiratory tract infections (48). Similarly, our cohort found that racial minority children had lower odds of receiving medical interventions, possibly due to implicit bias, parental expectations, and disparities in primary care access.

Indigenous children were the only group at higher risk of hospitalization in our study. Studies have reported increased risk of severe SARS-CoV-2 infection among Indigenous populations in Canada (49) and other countries (50–52), possibly due factors rooted in colonialism (e.g., overcrowding, and lack of access to primary care) (49,53). Our finding that other racial groups are at lower or equal risk of hospitalization compared to White children differs from existing literature (1,2,39,54,55). Most studies used broad racial categories, limiting direct comparisons. Additionally, none of these studies reported on children in Canada, where the healthcare system and local cultural influences may impact racial minority groups differently than what occurs in other countries. These findings highlight the importance of disaggregated racial data to uncover differences between racial minority groups. Further research is needed to understand factors influencing hospitalization in COVID-19 among children of various races and ethnicities in Canada and abroad.

Only Black children had lower odds of PCC than White children in our study. However, since racial minority children are more likely to test positive for SARS-CoV-2 (1,2,54,56), the absolute number of PCC cases may be higher among racial minority children. Some reports suggest that Black adults are more likely to experience long-term COVID-19 symptoms (57,58), but no studies have evaluated PCC across multiple racial groups. Systemic racism (59,60) and issues of distrust in healthcare may have led to the under-reporting of PCC symptoms like pain (61,62) and psychological symptoms (63) among racial minority groups, including Black children (34,35). Similarly, cultural differences may influence how racial minority families report chronic symptoms, and language barriers may hinder the reporting of PCCs among racial minority children. Therefore, our findings may also represent under-reporting of PCCs among racial minority children.

### Limitations

First, we did not collect socioeconomic data, which may be a confounding factor in the association between race and SARS-CoV-2 test positivity and outcomes (64). Second, there may have been sampling bias in our cohort, as we did not ascertain participants’ access to primary care. White children have better access to primary care in Canada (65), and may have been screened by their primary care provider. Thus, white children presenting to ED may have been sicker than their racial minority counterparts, increasing their rates of medical interventions and hospitalization (66). Third, we cannot distinguish the associations between race and medical interventions/hospitalization from the association between race and resource utilization. Fourth, some participants did not identify with the races in the self-reported questionnaire, highlighting the complex intersectionality of race, and the limitations of self-reported standards in Canada. Similarly, participants who identified with more than one race were grouped in a Multiracial category, despite a wide range of combinations encountered, limiting our ability to make meaningful conclusions for this group. There remains a need for guidance on how to best report and analyze individuals who identify as Multiracial. Fifth, we prioritized reporting across ten race categories, recognizing that racial minority groups are different from each other, as is the impact of COVID-19 on each of these groups. This limited the covariates we could adjust for, and our results do not account for other factors possibly impacting hospitalization and PCC. Sixth, medical intervention was analyses as a heterogenous composite outcome, to avoid over-testing, and our findings must be interpreted accordingly.

In conclusion, SARS-CoV-2 test positivity, receipt of medical interventions, hospitalizations, and PCC among Canadian children seeking ED care differ by race. It remains unclear whether these differences relate to healthcare seeking behaviors or systemic factors. This study highlights the need for additional work to understand the etiology of these differences in specific populations, and design policies to address health inequities.

## Supplementary Data

Supplementary data are available at *Paediatrics & Child Health* Online.

pxaf058_suppl_Supplementary_Table_1

pxaf058_suppl_Supplementary_Table_2

pxaf058_suppl_Supplementary_Figures_1-2
