# Supplementary material for: Racial disparities in children tested for SARS-CoV-2 at pediatric emergency departments: A prospective cohort study
Source: Paediatr Child Health. 2025 Aug 9;30(8):710–22. doi: 10.1093/pch/pxaf058 (PMC12718024; doi:10.1093/pch/pxaf058)
Supplement: pxaf058_suppl_Supplementary_Table_1 [file pxaf058_suppl_supplementary_table_1.docx]

**Supplemental Table 1:** Re-categorization of race from Statistics Canada and free text entries to CIHI reporting standards.

| **Group** | **Survey group selected** | **Written under Other** |
| --- | --- | --- |
| Black | Black | African American  African Canadian  Afro-  Congolese  Dominican  Eritrean  Ethiopian  Grenada  Guinean  Haitian  Jamaican  Malagasy (Madagascar)  Mauritian  Nigerian  Somalian  St. Lucien  Sudanese  West African |
| East Asian | Chinese  Japanese  Korean | Chinese  East Asian  Hong Kong  Mongolian  North Asian  Northeast Asian  Taiwanese  Tibetan |
| Indigenous | Indigenous | Blackfoot  Indigenous  Metis |
| Latin American | Latin American | Brazilian  Colombian  El Salvadorian and Ecuadorian  Hispanic  Latino  Mexican  Puerto Rican  South American |
| Middle Eastern | Arab  West Asian | Algerian  Arabic  Berber  Cabile  Egyptian  Iranian  Iraqi  Kurdish  Lebanese  Libyan  Magreb  Middle Eastern  Moroccan  North African  Omani  Palestinian  Persian  Syrian  Tunisian |
| Multiracial |  | Interracial  Biracial  Mixed  Black + East Asian  Black + East Asian + White  Black + Indigenous  Black + Indigenous + White  Black + Indigenous + South Asian + White  Black + Latin American  Black + Latin American + White  Black + Latin American + Middle Eastern + White  Black + Middle Eastern  Black + Middle Eastern + White  Black + Other race  Black + Other race + White  Black + South Asian  Black + South Asian + White  Black + Southeast Asian  Black + Southeast Asian + White  Black + White^1^  East Asian + Indigenous  East Asian + Indigenous + Southeast Asian + White  East Asian + Indigenous + White  East Asian + Latin American  East Asian + Latin American + White  East Asian + Middle Eastern  East Asian + Other race  East Asian + Other race + White  East Asian + South Asian  East Asian + South Asian + White  East Asian + Southeast Asian  East Asian + Southeast Asian + White  East Asian + White^1^  Indigenous + Latin American  Indigenous + Latin American + Other race + White  Indigenous + Latin American + White  Indigenous + Middle Eastern  Indigenous + Middle Eastern + Southeast Asian + White  Indigenous + Other race  Indigenous + South Asian  Indigenous + Southeast Asian  Indigenous + Southeast Asian + White  Indigenous + White^1^  Latin American + Middle Eastern  Latin American + Middle Eastern + White  Latin American + Other race  Latin American + South Asian  Latin American + South Asian + Southeast Asian  Latin American + South Asian + White  Latin American + Southeast Asian  Latin American + White^1^  Middle Eastern + Other race  Middle Eastern + Other race + White  Middle Eastern + Other race + South Asian  Middle Eastern + South Asian  Middle Eastern + South Asian + White  Middle Eastern + South Asian + Southeast Asian  Middle Eastern + Southeast Asian  Middle Eastern + Southeast Asian + White  Middle Eastern + White^1^  South Asian + Southeast Asian  South Asian + Southeast Asian + White  South Asian + Other race + White  South Asian + Other race  South Asian + White^1^  Southeast Asian + Other race  Southeast Asian + White^1^ |
| Other race |  | Afghanistani  African  Asian ^1^  Canadian  Caribbean  Central Asian  Euro-Asian  Guyanese  Israel  Jewish  Muslim  Tajikistani  Trinidad, Guyanese, Grenada  Turkish  Unknown Asian ancestry  Visible Minority  West Indian |
| South Asian | South Asian | Bangladeshi  East Indian  Indian  Indo-Canadian  Indo-Fijian  Guyanese Indian  Punjabi  Sikh  Sri Lankan |
| Southeast Asian | South East Asian  Filipino | Phillipino  Thai  Vietnamese |
| White | White | Armenian  Australian  Azerbaijan  Belarusian  Bosnian  British Isle  Caucasian  Croatian  Danish  English  European  European Hispanic  French  French Canadian  German  Greek  Irish  Italian  Kosovar  Macedonian  Mediterranean  Mixed European  Norwegian  Polish  Portuguese  Quebecois  Romanian  Russian  Scottish  Serbian  Slovakian  Spanish  Ukrainian  Western European and Eastern European  “Brazilian, Arabian, but identify as White” |
| Race unspecified |  | Correct  Did not want to disclose  Not comfortable to specify  Prefer not to say  Declined to answer  No  None  Not willing to answer  None  Prefer not to answer  Unknown  Unsure  (or all race/ethnicity questions skipped) |

^1^ Reclassified to minority race group in sensitivity analysis
