# Supplementary material for: Racial disparities in children tested for SARS-CoV-2 at pediatric emergency departments: A prospective cohort study
Source: Paediatr Child Health. 2025 Aug 9;30(8):710–22. doi: 10.1093/pch/pxaf058 (PMC12718024; doi:10.1093/pch/pxaf058)
Supplement: pxaf058_suppl_Supplementary_Table_2 [file pxaf058_suppl_supplementary_table_2.docx]

**Supplemental Table 2: SARS-CoV-2 vaccination eligibility and rates by age group in children 0-17 years tested for SARS-CoV-2 at 14 Canadian pediatric emergency departments between August 2020 and February 2022 and vaccination rates by age group in the general population at the end of the study period (February 2022).^1^**

|  | Ineligible for vaccination at index visit  N=6304 | Vaccination status unknown  N=116 | Unvaccinated at index visit  N=624 | Partially vaccinated at index visit  N=96 | Fully vaccinated at index visit  N=93 | Vaccination rates (≥1 dose) among Canadians in February 2022^2^ |
| --- | --- | --- | --- | --- | --- | --- |
| **0-4 years, n (%)**  **(n=5128)** | 5162 (100%) | 0 (0.0%) | 0 (0.0%) | 0 (0.0%) | 0 (0.0%) | 0% |
| **5-11 years, n (%)**  **(n=1408)** | 738 (51.9%) | 57 (4.0%) | 545 (38.4%) | 70 (4.9%) | 11 (0.8%) | 55% |
| **12-17 years, n (%)**  **(n=648)** | 404 (62.2%) | 59 (9.1%) | 79 (12.2%) | 26 (4.0%) | 82 (12.6%) | 85% |

^1^In Canada, Health Canada approved SARS-CoV-2 vaccination for children age 0-4 years in August 2022, for children 5-11 years in November 2021, and for children 12-17 in June 2021.

^2^Data from the Canadian general population was obtained at the following reference: Public Health Agency of Canada. Canadian COVID-19 vaccination coverage report. Ottawa: Public Health Agency of Canada; July 12, 2024. Accessed online on November 4th, 2024 at https://health-infobase.canada.ca/covid-19/vaccination-coverage/
