# Supplementary material for: Racial disparities in children tested for SARS-CoV-2 at pediatric emergency departments: A prospective cohort study
Source: Paediatr Child Health. 2025 Aug 9;30(8):710–22. doi: 10.1093/pch/pxaf058 (PMC12718024; doi:10.1093/pch/pxaf058)
Supplement: pxaf058_suppl_Supplementary_Figures_1-2 [file pxaf058_suppl_supplementary_figures_1-2.docx]

**Supplemental Figure 1:** Visual representation of race categorization for Multiracial participants who reported their race as White and minority groups in the primary (A) and sensitivity (B) analyses.

**Supplemental Figure 2:** Forest Plot demonstrating the odds of SARS-CoV-2 clinical outcomes for children of various racial backgrounds tested for SARS-CoV-2 in Canadian pediatric emergency departments between August 2020 and February 2022, when a sensitivity analysis of participants who self-identified as Multiracial (White and a racial minority group) are re-categorized to their minority group is performed^1,2^

^1^ Results for test positivity and medical interventions were obtained using multivariate regression model adjusting for age, sex, pre-existing conditions, attendance at social gatherings and school/daycare, mask wearing, duration of symptoms prior to presentation, VoC period, and ED site. Results for hospitalization were obtained using a multivariate regression model adjusting for age, sex, and ED site. Results for PCC were obtained using multivariate regression model adjusting for age

^2^Participants were excluded from the test positivity and medical intervention models due to missing values for the following covariates: attendance at large social gatherings (n=15), attendance at in-person school (n=4), mask wearing (n=2), symptom duration (n=1).
